# Disrupted knee – disrupted me: a strenuous process of regaining balance in the aftermath of an anterior cruciate ligament injury

**DOI:** 10.1186/s12891-022-05252-6

**Published:** 2022-03-26

**Authors:** Josefin Karlström, Maria Wiklund, Eva Tengman

**Affiliations:** 1 Department of Community Medicine and Rehabilitation, Section for Physiotherapy, Umeå University, Umeå, Sweden; 2 Umeå School of Sport Sciences, Umeå University, Umeå, 90187 Sweden

**Keywords:** ACL, Physiotherapy, Qualitative Research, Interviews, Coping strategies, Fear of re-injury, Rehabilitation, Psychological impairments

## Abstract

**Background:**

Individuals describe both short and long term consequences after an anterior cruciate ligament (ACL) injury. Functional impairments are well documented while psychological, social and contextual factors need to be further investigated. By the use of a qualitative method incorporating a biopsychosocial lens, we aimed to explore individuals’ experiences of living and coping with an ACL rupture with a specific focus on experiences significant to overall life, activity in daily living and physical activity more than one year after injury.

**Methods:**

Twelve participants were chosen strategically by a purposive sampling. Four men and eight women (19–41 years) with an ACL rupture 2–25 years ago, were included. Semi-structured interviews were used and analysed with qualitative content analysis.

**Results:**

The results consisted of one overarching theme: ‘A strenuous process towards regaining balance’ which built on three categories ‘Disrupted knee’, ‘Disrupted me’ and ‘Moving forward with new insights’. The overarching theme captures the participants’ experiences of a strenuous process towards regaining both physical and mental balance in the aftermath of an ACL injury. The results illuminate how participants were forced to cope with a physically ‘disrupted knee’, as well as facing mental challenges, identity challenges and a ‘disrupted me’. By gradual acceptance and re-orientation they were moving forward with new insights – although still struggling with the consequences of the injury.

**Conclusions:**

Individuals with an ACL injury experience both physical, psychological, and social challenges several years after injury. In addition to the functional impairments, diverse psychological, social and contextual ‘disruptions’ and struggles may also be present and influence the rehabilitation process. It is important that physiotherapists identify individuals who face such challenges and individually tailor the rehabilitation and support. A biopsychosocial approach is recommended in the clinical practice and future studies focusing on psychosocial processes in the context of ACL rehabilitation are warranted.

## Background

Anterior cruciate ligament (ACL) rupture is a common injury to the knee in physically active individuals [1] and especially among those who participate in pivoting sports, e.g. soccer, handball, or floorball. ACL ruptures are considered as one of the most severe sports-related injuries [2] and often lead to remaining functional impairments, difficulties returning to sport and for many the end of an athletic career. Returning to sport is usually the primary concern for athletes and meta-analyses found that only 60–65% of ACL-injured return to their pre-injury activity level [3, 4]. Reasons described for not returning to sports include functional impairment and psychological factors such as fear of re-injury and psychological readiness [4–7].

Long-term consequences are another important perspective of an ACL rupture. Common consequences are functional impairments, lower physical activity level and a high risk of knee osteoarthritis (OA) [8–10]. Important experiences when it comes to having a high quality of life five to twenty years after ACL reconstruction are physical activity preference, lifestyle modification, adaptions and acceptance, and fear of re-injury. The most important factor for quality of life is maintaining a physically active lifestyle. Fear of re-injury is also described in the long-term perspective [11].

Functional impairments such as impaired strength, balance, and jump capacity after an ACL rupture are well described. A recent review regarding psychosocial factors after a sport-related knee injury (including both ACL and other knee injuries) concludes that it is also important to consider psychological, social and contextual aspects as essential components in rehabilitation after a sport-related knee injury [12]. That review also showed a predominance of studies with quantitative methods and considerably fewer included studies with qualitative methods describing the individuals’ experiences. Only 30% (*n* = 23) of the included studies had a qualitative method or mixed method and of those 16 studies were focusing on ACL-injury [12]. This highlights the need of qualitative research with a biopsychosocial perspective investigating individual experiences.

Qualitative research discloses, for instance, psychological aspects such as negative emotions, frustration and anxiety [13–15], and that handling these negative emotions may be important for recovery. Other important aspects for recovery, described in qualitative research, are coping strategies and motivation. Active coping strategies and “taking control” are important during rehabiliation of ACL injuries [13, 16, 17] as well as establishing realistic expectations and goals for the rehabilitation [14, 18]. Another important psychological aspect is the identity change or loss of identity [15, 19, 20]. In a study by Scott et al. (2018) individuals described that the ACL injury had irrevocably changed them and their outlook on life [19]. Further, several of the qualitative articles described the importance of social support from physiotherapist, coaches and family when recovering after an ACL injury [13, 14, 16–18, 21]. Qualitative research can thus reveal important experiences that are difficult to measure quantitatively.

This study seeks to address several knowledge gaps. Since existing research focuses primarily on the early stages of rehabilitation mainly in athletic populations, this study focuses on later stages and post-rehabilitation in both athletic and non-athletic individuals. Because most previous studies are quantitative, we chose to use qualitative methodology. By highlighting the consequences of an ACL injury from the injured person’s perspective, knowledge and understanding for the remaining impairments, psychological consequences and coping strategies can increase which may also impact the clinical practice. Therefore, by the use of a qualitative method incorporating a biopsychosocial lens, we aimed to explore individuals’ experiences of living and coping with an ACL rupture with a specific focus on experiences significant to overall life, activity in daily living and physical activity more than one year after injury.

## Material and methods

### Study design

The present study has a qualitative and inductive research design. Semi-structured interviews were used and analysed with qualitative content analysis [22].

### Participants

Twelve individuals with an ACL rupture were included in this study. They were chosen strategically through purposive sampling from northern Sweden and from an orthopaedic clinic in that area. The purposive sampling aimed for variation for sex, age, physical activity level, time since injury, one or several ACL injuries and reconstructed or non-reconstructed treatment. Inclusion criteria were age between 16–60 and an ACL rupture more than one year ago. The exclusion criteria were inability to speak or understand the Swedish language. Knee function was assessed by the Lysholm knee score [23] and Knee injury and Osteoarthritis Outcome Score (KOOS) [24] were 0 = worst and 100 = optimal knee function. Knee-specific activity level was assessed according to the Tegner activity scale where 0 represents sick-leave due to the knee, and 10 represents practicing competitive sports on national or international level in sports that are demanding to the knee joint e.g. soccer [23]. Tampa Scale for Kinesiophobia (TSK-sv) scale was used to assess fear of movement/re-injury. The total score ranges from 17 to 68, with higher scores indicating greater fear of movement [25]. The project was approved by the Regional Ethical Review Board in Umeå, (Dnr 2015/67–31), and followed the principles of Declaration of Helsinki.

### Data collection

Semi-structured interviews were used for data collection. An interview guide was used and consisted of mainly open questions. WHOs International Classification of Functioning, Disability and Health (ICF) [26] informed the interview guide, focusing on participants’ experiences of 1) body function and structure, 2) activity, 3) participation, 4) environmental factors, and 5) personal factors. During the development of the interview guide, two pilot interviews were conducted with people with experience of ACL-injury, which guided the research group to refine the research questions. The initial open questions were: “Can you tell me about your experiences of living with an ACL rupture?” and “How do you experience that your life has been affected by your ACL rupture?” Various open-ended follow-up question covered experiences within these all five areas of ICF.

The interviews were conducted by the first author (J.K) who is a physiotherapist with clinical experience of rehabilitation of individuals with ACL injury. The interviews were conducted individually face to face (*n* = 11) or by telephone (*n* = 1), lasted between 28 and 38 min, and took place at a location proposed by the interviewer or the participants. All interviews were audiotaped and transcribed verbatim.

### Data analysis

Qualitative content analysis was used [22]. The analysis started with identifying meaning units which were condensed and provided with codes which captured the manifest content. Codes were then grouped into sub-categories and categories based on similarities and differences in content. Finally, one theme was formulated which captured the latent content running through the codes and categories. In this study, trustworthiness was enhanced by triangulation between researchers with different competencies and perspectives [22]. During this process, emerging codes, categories and themes were continuously discussed and agreed upon. Trustworthiness was also ensured by peer-debriefing with colleagues experienced within the field. The computer software Open Code 4.03 was used to support the analysis (ICT Services and System Development and Division of Epidemiology and Global Health. Umeå University).

## Results

Eight women and four men participated in the study and were aged 19 to 41 (median 32) years of age. The median time since ACL rupture was 4 years (range 2–25 years). Some participants had concomitant injuries such as meniscus tear, medial or lateral collateral ligament injury, or cartilage injuries and one participant had bilateral ACL injuries. Four participants also had re-ruptured the reconstructed ACL. Ten participants had undergone an ACL reconstructive surgery, one was waiting for surgery and one had a non-surgical treatment with only physiotherapy. The participants had a variation in self-reported knee function, physical activity level and fear of movement/re-injury (Table 1).

**Table 1:** Background information of the participants

Participant	Sex	Age (years)	Years since injury	ACL-R	KOOS Pain	KOOS Symptom	KOOS ADL	KOOS Sport /Rec	KOOS QOL	Lysholm	Pre Tegner	Current Tegner	TSK-sv
1	M	34	12	Yes	97	86	97	56	75	69	7	5	33
2	F	32	12	Yes	92	82	99	95	69	95	6	4	32
3	M	29	7	Yes	67	64	88	45	44	89	9	4	36
4	F	31	4	Yes	89	82	100	60	44	85	6	5	26
5	M	41	25	Yes	44	25	65	5	13	44	10	5	41
6	F	24	4	Yes	89	75	94	90	50	81	7	6	51
7	F	37	14	Yes	100	100	100	85	69	90	10	7	22
8	F	27	2	No	69	50	90	15	31	59	6	4	42
9	F	35	3	No	86	93	90	45	56	68	5	4	23
10	F	19	2	Yes	100	79	100	70	56	99	10	10	36
11	M	33	15	Yes	53	64	71	45	31	53	9	4	47
12	F	23	3	Yes	83	75	100	70	56	73	8	7	21
N/ Median (range) (Min–max)	4 M/8F	32 (22) 19–41	4 (23) 2–25	10Y/2 N	88 (55) 44–100	77 (75) 25–100	96 (35) 65–100	58 (90) 5–95	53 (63) 13–75	77 (55) 41–99	7.5 (5) 5–10	5 (6) 4–10	34.5 (30) 21–51

*M* Men, *F* Female, *ACL-R* Reconstruction surgery, *KOOS* Knee injury and Osteoarthritis Outcome Score, *Lysholm* Lysholm knee score, *Tegner* Tegner activity scale, *TSK-sv* Tampa Scale for Kinesiophobia

### A strenuous process towards regaining balance

**Fig. 1:**
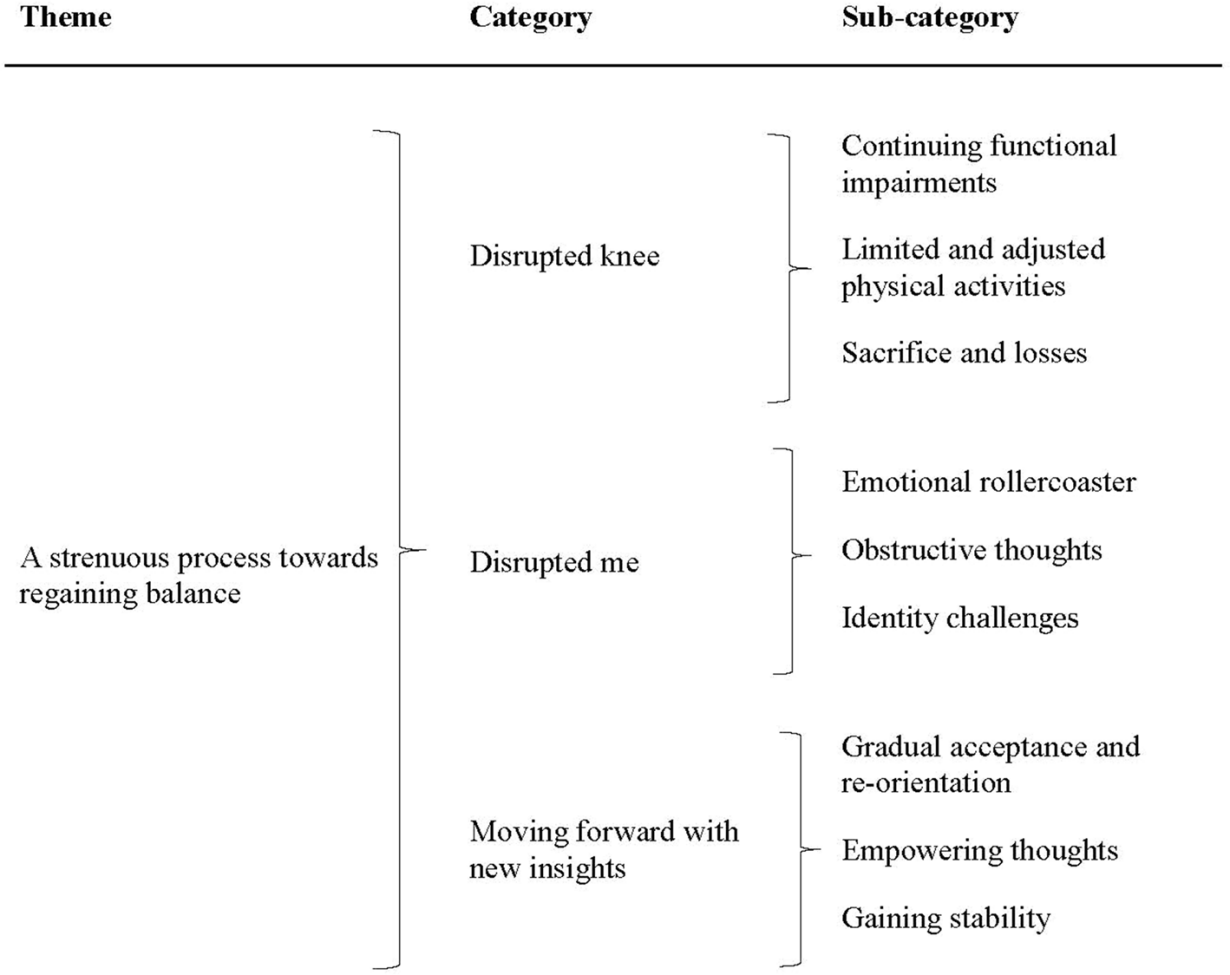
Overview of results from the qualitative content analysis with the over-arching theme, categories and sub-categories

The qualitative analysis resulted in one overarching theme ‘A strenuous process towards regaining balance’ and three categories: ‘Disrupted knee’, ‘Disrupted me’ and ‘Moving forward with new insights’ with interrelated sub-categories (see Fig. 1). The overarching theme captures participants’ experiences of a strenuous process towards regaining balance in the aftermath of an ACL injury. It refers to participants’ process of regaining physical, mental and social balance after the loss; and how they by gradual acceptance and re-orientation were moving forward with new insights – although still struggling with the consequences of the ACL injury. The theme refers to their struggle of making adjustments and regaining knee function and overall wellbeing after the injury. Moreover, it captures how participants were forced to cope with a physically ‘Disrupted knee’, facing mental challenges, identity challenges and a ‘Disrupted me’.

### Disrupted knee

The category ‘Disrupted knee’ comprised participants’ experiences of ‘Continuing functional impairments’, ‘Limited and adjusted physical activities’, and ‘Sacrifices and losses’ made.

#### Continuing functional impairments

Continuing functional impairments such as instability, pain, stiffness and weakness were still experienced by the participants more than 1 year (median 4 years) after the ACL injury. The instability was perceived during physical activities like jumping and everyday activities such as walking, especially on slippery surfaces, which could cause anxiety. For some, the knee was well-functioning except in rotations. Pain could appear during, after, or the day after activities – ranging from mild to severe pain. The pain could also be perceived as extending to other parts of the body:

‘Now it's not just that the knee hurts, but it can hurt everywhere’. (P3)

Knee joint stiffness was experienced during physical activities and also at rest following activities. Due to the stiffness, it took longer to perform an activity, which was perceived as a limitation.

‘The largest problem is that you get much slower and feel stiff’. (P1)

Loss of muscle mass, reduced muscle strength, problems from the graft donor area, and remaining numbness on the shinbone were also described. Various muscle problems were expressed:

‘The biggest problem is the back of the thigh, reduced strength, and I often pull the muscle’. (P11)

#### Limited and adjusted physical activities

Due to continuing functional impairments, participants described limiting and adjusting their physical activities in everyday life, leisure, sports, and work situations. They avoided some activities and situations. They described having had high physical activity levels before the injury, which they had not regained after the injury.

’Earlier I worked out a lot and that has been limited the most.’ (P1)

These limitations were primarily due to reduced knee function, exemplified by:

’…jumping, sideway movements, but it can even be everything from, well, quick turns, starts and stops, sprints...it’s impossible.’ (P4).

Everyday life activities were still affected, such as standing, squatting, sitting, navigating stairs, walking downhill or on slippery surfaces, and when sleeping with the leg in a specific position.

‘I'm a little more careful when I feel that [insecurity] on slippery surfaces, or if it is steep downhill, where I feel a bit unstable, so I may go a little slower’ (P7)

To manage, participants had to become more mindful and cautious, and modify their activities. Some wore a brace during physical activities, and tried to allocate time for continuous rehabilitation training. Some activities and situations were avoided.

#### Sacrifices and losses

Sacrifices and losses were experienced due to the injured knee. Sacrifices were made to maintain a functioning knee and to participate in activities. Playing with their own children was also perceived as difficult. Participants had to make choices in their social life that also involved and influenced others, including friends and family members. These choices could concern participation in activities – or not:

‘For example, if you should go with friends and do things, like play softball – you say no. Because, you do not want it [ACL] to tear again’ (P8).

‘When my wife want to do activities like that [walk and stand for a long time], we were hindered because of my knee injury’. (P1)

Participants suggested that they could not be as spontaneous as before the injury.

‘It is mostly when you are going to do something, maybe if you are a group of friends and you are going to do something spontaneously’ (P3)

A shared experience among some participants were that they still tried to engage in physical activities so that they did not miss important social interactions although they knew that pain would be a consequence. However, several of them chose most often to decline an activity to avoid placing their knee and future function at risk.

### Disrupted me

The category of ‘Disrupted me’ refers to experiences of an ‘Emotional rollercoaster’, ‘Obstructive thoughts’, and ‘Identity challenges’. As the used metaphor of an emotional rollercoaster implies, the aftermath of the knee injury included psychological imbalance and a ‘disrupted me’. Multiple and mixed feelings were described, as well as a loss of former social identity – both in the eyes of themselves or others.

#### The emotional rollercoaster

The ‘emotional rollercoaster’ comprises discrepant feelings such as disappointment, guilt, sorrow, hopelessness, sadness and hope. Disappointment was articulated because of limitations and not being able to participate in activities as they wished. The feeling was described that exercise could be both helpful or a hindrance. Guilt emerged in different ways. Some participants felt guilty and as a ‘burden’ to others. For example, consideration from friends became a negative feeling instead of being perceived as encouragement. In other cases guilt and anger were directed towards themselves, for instance if they had pushed themselves too hard and risked their knee.

‘Then I’m most annoyed at myself, perhaps if I have pushed myself too hard or done something that I really shouldn’t have done…because I couldn’t wait, just wait a little more” (P2)

Sorrow was expressed as feelings of frustration and sadness, for example when realizing that they were unable to achieve the pre-injury level of physical active, with limited spontaneity. The aftermath of the injury also brought a sense of hopelessness due to the limitations in sports and everyday life, for example when no longer being able to exercise or run at high intensity. A feeling of sadness emerged when thinking about quitting their sport, being forced to stand on the sideline, or to no longer being asked to participate. The sadness also embraced missed social community.

‘It's a little hard to let go of my sport completely. I had perhaps wanted to continue with my sport’ (P3)

Although doubting, participants still hoped to regain the same level of activity as before the injury, or to regain full function of the knee. As one participant expressed;

‘I wish, that it [the knee] will work, like it did before’. (P4)

#### Obstructive thoughts

Obstructive thoughts were experienced about the injury and surgery. Participants’ described that their mental state and ‘mindset’ sometimes became an obstacle, for example through anxiety and fear in activities that challenged the affected knee. A ‘mental block’ was described, for example expressed as ‘it is always in the back of my mind’ (P10). Thus, lack of trust and uncertainty of the knee function emerged, including hesitance to fully use the knee.

A general sense of fear of movement was shared by the participants and primarily related to the risk of re-injury. It was talked of as a reason for decreased physical activity level and avoiding some activities, and was particularly tied to activities which could risk hurting the knee again. Often the fear was more intense than the actual pain.

‘It is perhaps more in the head [mentally], which holds me back. I am afraid that it [ACL] not will hold, although it may do so.’(P3)

‘Yes, but I'm probably a little extra scared and carful with my knee…. probably in almost all situations’ (P9).

The fear was combined with being angry and sad in situations where the knee was limiting function. This anxiety was present in everyday life, at work and in various physical activities. As expressed below, fear to re-injury due to weakness in the hamstrings led to panic:

‘I panic because it [the rupture and its consequences] was so darn difficult, I don’t want to do that again, I would rather break all of my bones…’. (P6)

Further, there were obstructive thoughts about the future, including anxiety about further surgeries and need of knee replacement due to osteoarthritis.

‘I’m aware of the high risk of forthcoming arthrosis …and it feels hard to go through long rehabilitation periods in the future’ (P9).

Thus, the obstructive thoughts also concerned the long-term perspective, both regarding potential re-injury and restricted activities.

#### Identity challenges

Identity challenges implied a sensed ‘loss of identity’, as the injury not only affected the knee but the whole person and personality, often tied to the sacrifices made to manage the overall life.

‘It was like all of me and my entire identity… like disappeared…’ (P4)

Participants’ view of themselves had changed, and they felt less ‘needed by others’ than pre-injury, as they were now accomplishing less. They had a feeling of becoming a ‘coward’ and old. Increased body weight and reduced fitness could also be part of this change sense of self:

‘Before I was like, active, physically and yes, well trained and after that, after the injury I gained weight and was more sedentary and stuff …so it has definitely been different’ (P1).

In the aftermath of the injury they had tried to find new activities, which was a challenge as they could not change their personality and interests too much.

Experiences of being changed in the eyes of others was mainly connected to the inability to engage in sports and all the physical activities that had formed part of their (former) social life and relationships. However, the change of identity was also perceived as positive, as the injury had, in a sense, strengthened them as a person and they were more appreciative of what they had:

‘It has strengthened me in a way…that I have started, well, appreciate what I have now when I’m back. Like that I’m lucky to be injury-free. ’ (P10)

Such reflections could reflect developing a new more positive mindset for life.

### Moving forward with new insights

This category refers to experiences of ‘Gradual acceptance and re-orientation’, ‘Empowering thoughts’ and ‘Gaining stability’. It captures participants’ gradual acceptance of the injury and its consequences – and describes how they developed coping strategies to regain stability in the knee, in themselves and in life as a whole. Despite remaining functional impairments, there was a shared sense of regaining stability and finding a way to be satisfied with the ‘new’.

#### Gradual acceptance and re-orientation

A gradual acceptance of the ACL-injury and its consequences was perceived, but also re-orientation. Acceptance of ‘standing by the side’ during sports and physical activities was described, and now they felt that ‘it doesn’t matter’ and was not that tough anymore.

‘Now, I've learned to handle and accept it, even though I'm sad that I can’t do everything I want’. (P5)

Re-orientation had been crucial in the process of acceptance, both for themselves and for those around them.

‘I had to find new activities that in some way could replace the old ones, that's probably the biggest challenge. And then it has been tough to accept that it [ACL] is torn, but it is not like I die of it…because this is something that very MANY people live with [ACL injury]. I'm not the first and will not be the last one. So to be able to accept, that it actually works. There are some things that just need to be adjusted.’ (P4)

Thus, participants had learned to deal with the limitations in various ways and had become more aware of their ways of coping. They had changed activities and adapted to their situation of living with an ACL injury by finding solutions to their limitations. They described that exercise was fun again, or accepted the loss of some activities. Tough times were handled by seeing them as transition periods.

#### Empowering thoughts

Empowering thoughts and motivation were key for successful rehabilitation. Participants reflected that the mental aspects were equally important as the physical when coping with their ACL injury.

‘My strength is that I have had motivation the whole time’. (P10).

When it came to dealing with the actual injury, their interest in physical activities was crucial, and they perceived a motivation to exercise more today than before the injury*:*

‘In this case, positive, that is, I have more of an incentive to exercise’. (P12)

For some, the attitude to be equally physically active after the injury as before was experienced as facilitating. Feelings of regaining confidence and no longer feeling anxious about knee function contributed to trust:

‘I don’t think it is a big problem [living with an ACL injury]’ (P7)

‘If it happens again, then it happens again. You cannot walk around being worried... It will hold. I am fully recovered’ (P10)

If a re-injury would occur, they believed that they now were better prepared for emotions that may arise, as they now were ‘mentally tougher’. The reclaimed knee function and recovery helped them to not feel as mentally ‘down’ as before. They were happy to cope with everyday activities again and not to worry.

#### Gaining stability

Gaining stability refers to the strenuous process of regaining knee function, and activity level as well as preventing re-injury. However, this required a substantial amount of both physical exercise and mental effort which was not without challenges. A sense of a re-claimed knee function and activity level emerged with a feeling of regaining stability. Crucial for regaining stability was to feel ‘ownership’ of the rehabilitation.

‘Otherwise it’s very much up to yourself to get this [rehabilitation]’. (P11)

Participants had gone from high physical activity (pre-injury) to a lower level. Some of them had returned to higher level again, even their pre-injury sport albeit not necessarily at the same level. Some had made ‘comeback’ to their sport as a coach. Now they knew their bodies better and were ‘smarter and listened to their body’ (P10). They also thought they had become more accommodating to themselves.

‘I think it's a pretty good experience to have gone through a long period of rehab or injury and be able to get back. Just having been through it and feel the emotions that you go through is probably quite meaningful. I definitely feel I have learned a lot, both about how to find motivation for myself and what I do when I don’t have any motivation, …and how I behave and so on.’ (P12)

Despite the strenuous process of overcoming physical, psychological, and social ‘disruption’, the participants experienced that they had learned new things about themselves and gained ‘new opportunities and perspectives’. This had also ‘opened their eyes’ of life outside sports.

‘I realised that – oh, there's a life outside’ (P10).

However, regaining balance had been – and could still be – a struggle, both physically and mentally.

## Discussion

This study provides valuable and novel insights to existing knowledge by synthesising experiences of the role of functional impairments, psychological and social aspects after an ACL injury, which indicates that the whole person is affected and not only the knee. The main result captures participants’ experiences of a strenuous process towards regaining balance in the aftermath of an ACL injury, and the participants’ struggle of making adjustments and regaining knee function and overall wellbeing after the injury.

The category ‘Disrupted knee’ refers to participants’ experiences of functional impairments such as instability, pain, and reduced strength despite rehabilitation and several years (median 4 years range 2–25 years) after the ACL injury. This is in line with other literature reporting both short and long term physical impairments [8, 9, 11, 27]. In addition to existing knowledge, the present study highlights the experiences of struggling with these consequences and how they were limited in both physical activities, everyday life, and work situations. The participants ranged from athletic to non-athletic with high pre-injury activity levels (supported by the rating of Tegner activity scale, Table 1), and they had not achieved the same level post-injury. This is in line with results from other research showing difficulties to return to pre-injury activity level [3, 4] and some had adopted a physically inactive lifestyle [6, 28, 29]. Despite being several years after injury, the participants described that some activities and situations were still avoided and that they had to become more mindful and adapt their activities. Also, other qualitative studies have shown similar short- and long-term experiences [11, 30]. Our results emphasise the importance of guidance and support from clinicians (e.g. physiotherapists) to adjust or find new activities that are feasible during rehabilitation – but also in the long term perspective throughout various stages of their life.

Our results reveal that the physical injury (disrupted knee) had far-reaching psychological, emotional and social consequences. Negative emotions were evident as disappointment, guilt, sorrow, hopelessness, sadness and hope, adding to commonly reported frustration and anxiety [13–15, 31, 32]. Such psychological experiences as well as managing these challenges may differ between male and female athletes [31]. These negative feelings and psychological aspects may play an important role for recovery and psychological factors may predict outcomes after an ACL-R [33]. Even though a recent consensus statement article concludes that psychological factors play an important role in return to sport, it remains unclear if psychological scales can be used to capture this and improve the rehabilitation process [34]. Thereto, a study by Piussi et al. 2021 suggested that physiotherapists express insufficient knowledge of how to address psychological impairments during rehabilitation [35]. Our result can provide guidance of important aspects for screening, e.g. psychological distress and fear of movement, but also enhance communication skills in consultation [36, 37]. Further research should address psychological challenges during the rehabilitation process and how professionals can respond to these, since structured psychological support may be beneficial for recovery and re-orientation.

Our results point to obstructive thoughts related to fear and anxiety tied to the injury and to activities that challenge the affected knee. Fear of re-injury is a commonly reported reason for individuals not to return to sport [4–7]. The present study also contribute insights on how fear of re-injury can be experienced in several situations in everyday life, leisure time and work. However, a fear of re-injury is not completely unjustified since systematic reviews and meta-analysis show that individuals who return to a high activity level after primary ACL-R have a high risk of suffering a secondary ACL injury to their ipsi- or contralateral knee [38, 39]. Moreover, our participants raised a fear of long-term consequences and the awareness of the risk of OA after the injury, which is in line with other studies [16]. High fear of re-injury has also been associated with poor knee-related quality of life after ACL-R [29]. In the present study fear of re-injury/movement was clearly described in the interviews but also seen, for some participants, in high ratings of TSK-sv. Those with high TSK-sv score also seemed to have lower self-reported knee function (Table 1). Persons who over-come re-injury fears are more likely to continue sport participation and describe a more satisfactory quality of life [11]. As in other ACL populations, knee function and degree of fear of re-injury vary within the group. Therefore, it is important that physiotherapists can identify those with high fear of re-injury/movement. Rehabilitation needs to be individually tailored and those with high fear of re-injury/movement probably need more support and guidance, as well as in finding strategies that can help them in everyday life in the long term. Approaches such as cognitive behaviour therapy [36] and cognitive functional therapy [37], including graded exposure may be useful. Those have been effective in reducing fear of movement and avoidance in other musculoskeletal disorders, often related to pain e.g. low back pain [36, 37]. These approaches may have implications for coping with fear of re-injury/movement and improving psychological wellbeing after and ACL injury. Further research is needed in the context of ACL rehabilitation to address the multifaceted physical and psychological aspects.

Importantly, our results reveal a sensed ‘loss of identity’ and social ‘disruption’, as the injury not only affected the knee but the whole person and personality as quoted ‘It was like all of me and my entire identity… like disappeared…’. Likewise, other qualitative research has raised an identity change, loss of identity or disruption of individuals’ ‘athletic identity’ [15, 19, 20, 31], which even changed them and their outlook on life [19]. In addition to earlier research, the participants in the present study expressed that the injury had a negative impact on their social life, for example on how they chose to ‘pass’ activities and that even playing with their own children was perceived as difficult. This can be interpreted as an experienced loss of social context and sense of coherence [40]. Social support from physiotherapist, coaches and family when recovery after an ACL injury is needed [13, 14, 16–18, 20, 21]. Social support has shown to be positively predictive of knee symptoms and rehabilitation compliance [33]. In our study, it can be presumed that participants also reclaimed some of their sense of (social) coherence when they, after re-orientation, gradually discovered the ‘life outside sports’.

The category of ‘Moving forward with new insights’ emphasises participants’ gradual acceptance of the injury and its consequences – including how they developed coping strategies to gain new stability in the knee, in themselves and in life as a whole. The results of ‘Gradual acceptance and re-orientation’ illuminate the experience going through a crises, but that it was the re-orientation the participants talked about the most. Re-orientation has proved to be an important part of accepting the injury, where they found new activities adjusted to the limitations and remaining problems. Also another qualitative study by Fjellman-Wiklund et al. (2021) present a similar core category ‘Re-orientation towards acceptance’ [30]. Acceptance can thus be viewed as central to re-orientation and recovery. This is in line with the definition of acceptance in Acceptance and Commitment Therapy (ACT) which involves recognition and acceptance of e.g. thoughts, emotions, and bodily sensations in the relation to goals and actions [41]. According to Kortte el al. (2009), higher levels of acceptance, measured by the Acceptance and Action Questionnaire, relate to higher levels of hope, positive affect, and wellbeing in individuals undergoing medical rehabilitation following e.g. orthopaedic surgery, and amputation, while lower acceptance has been related to more severe depression and negative affect [42]. For individuals with an ACL injury who struggle with acceptance and re-orientation, ACT may possibly be part of the rehabilitation.

Empowering thoughts and motivation were described as key factors for successful rehabilitation. Participants reflected that the mental aspects were equally important as the physical when coping with their ACL injury. Active coping strategies and ‘taking control’ are important during rehabiliation of ACL [13, 16, 17], while lack of strategies and loss of motivation are described as hindering factors [16, 17]. Active coping strategies have been described to be associated with greater motivation and a lack of self-motivation is described decreasing adherence of training [21]. Therefore, active coping strategies need to be addressed in rehabilitation.

Despite the challenges, limitations and continuing functional impairments, a sense of a re-claimed knee function and activity level emerged with a feeling of regaining stability. Crucial for regaining stability was to feel ‘ownership’ of the rehabilitation. This feeling may be tied to a combination of self-efficacy and locus of control. Self-efficacy is defined as a belief in one´s ability to succeed in a particular situation or execute actions, while locus of control is more of belief in the relationship between action and outcome; feeling like one has control. Both aspect seems to have an important role in recovery following a ACL injury [43]. Our study emphasises the importance of gaining stability by positive experiences such as learning new things about oneself leading to ‘new opportunities and perspectives’.

The present study has both methodological strengths and shortcomings. A major strength of the study was the sample variation, with variation in sex, age, years since injury, pre- and current activity level, self-reported knee function, and fear of movement/re-injury (see Table 1). Even though the sample size was limited, the data material was rich. To facilitate transferability, detailed description was provided of the context, sampling procedure and characteristics of participants, the data collection and the data analysis process. A rich presentation of the result together with quotations further enhanced the transferability and credibility. To enhance trustworthiness in interpretation of data, triangulation between authors with different perspectives was performed, as well as peer-debriefing [22], which also deepened understanding of the content in the interviews. Member-check was not performed of practical reason, although this would have been valuable. To ensure neutrality the authors were not involved in the participants’ rehabilitation. A potential bias in data collection and interpretation was that all three authors are physiotherapists and women, with experiences of rehabilitation of ACL injuries. However, it could also been a strength. Results from qualitative research are context-dependent [22] and considering a relatively small sample, from a single geographic area in Sweden the transferability of the findings might be limited. However, as pointed to in the discussion of the findings of an impact on the whole person, not only the knee, may be transferable to understand similar experiences for ACL injured. Nevertheless, more qualitative research is needed in additional subgroups and contexts.

## Conclusion

In terms of a ‘disrupted knee’ and a ‘disrupted me’, the results of this study display individual experiences of a strenuous process of regaining balance in the aftermath of an ACL injury. This highlights how regaining balance had been – and could still be – a struggle, both physically and mentally, and that the ACL injury, ‘is always in the back of my mind’ despite several years since the injury. Based on our results, professional support may be needed in rehabilitation of functional impairment as well as support of psychological, social and contextual ‘disruptions’ that influence the rehabilitation process. Therefore, it is important that physiotherapists can identify individuals who face such challenges, for example by using screening tools for psychological distress and fear of movement. Based on that, rehabilitation and support should be individually tailored. Individuals may also need support in adjusting activities and finding new activities that are feasible during rehabilitation but also in the long term. A biopsychosocial approach is recommended in the clinical practice, when meeting individuals with an ACL-injury. Future studies focusing on psychosocial processes in the context of rehabilitation are warranted, including development of guidelines for tailored psychological and social support.

## Data Availability

The data (interviews) analysed during the current study are not publicly available due anonymity of the participants but are available from the corresponding author on reasonable request.

## Abbreviations

ACL: Anterior cruciate ligament
ACT: Acceptance and Commitment Therapy
OA: Osteoarthritis
